# In-Depth Characterization of EpiIntestinal Microtissue as a Model for Intestinal Drug Absorption and Metabolism in Human

**DOI:** 10.3390/pharmaceutics12050405

**Published:** 2020-04-28

**Authors:** Yunhai Cui, Stephanie Claus, David Schnell, Frank Runge, Caroline MacLean

**Affiliations:** 1 Department of Drug Discovery Sciences, Boehringer Ingelheim Pharma GmbH & Co KG, 88397 Biberach, Germany; david.schnell@boehringer-ingelheim.com (D.S.); frank.runge@boehringer-ingelheim.com (F.R.); 2 Department of Drug Metabolism and Pharmacokinetics, Boehringer Ingelheim Pharma GmbH & Co KG, 88397 Biberach, Germany; stephanie.claus@boehringer-ingelheim.com (S.C.); caroline.maclean@boehringer-ingelheim.com (C.M.)

**Keywords:** Caco-2, EpiIntestinal, first-pass, P-gp, BCRP, drug transporter, CYP3A4, UDP-glucuronosyltransferase, carboxylesterase, oral availability

## Abstract

The Caco-2 model is a well-accepted in vitro model for the estimation of fraction absorbed in human intestine. Due to the lack of cytochrome P450 3A4 (CYP3A4) activities, Caco-2 model is not suitable for the investigation of intestinal first-pass metabolism. The purpose of this study is to evaluate a new human intestine model, EpiIntestinal microtissues, as a tool for the prediction of oral absorption and metabolism of drugs in human intestine. The activities of relevant drug transporters and drug metabolizing enzymes, including MDR1 P-glycoprotein (P-gp), breast cancer resistance protein (BCRP), CYP3A4, CYP2J2, UDP-glucuronosyltransferases (UGT), carboxylesterases (CES), etc., were detected in functional assays with selective substrates and inhibitors. Compared to Caco-2, EpiIntestinal microtissues proved to be a more holistic model for the investigation of drug absorption and metabolism in human gastrointestinal tract.

## 1. Introduction

Despite the recent innovations in drug delivery, oral administration remains the major route of drug administration. Understanding the drug absorption and metabolism in the intestine is thus essential for drug development. To date, several in vitro or ex vivo models are available for the evaluation of drug absorption and metabolism in human intestine. The Caco-2 cell culture model, e.g., is considered the gold standard in vitro model for studies of drug absorption, although there are limitations of this model with regards to drug metabolism [1,2]. Although a number of drug metabolizing enzymes (DME) have been identified in Caco-2 cells, including UDP glucuronosyltransferases (UGT) [3] and carboxylesterases (CES) [4], cytochrome P450 3A4 (CYP3A4), the major drug metabolizing enzyme in human intestine and liver is missing in Caco-2 cells [5]. Alternatively, sections of human intestinal tissue or mucosal biopsy mounted in Ussing chamber can be used to study drug absorption and metabolism in human intestine [6]. The advantage of this method is the combined measurement of permeability/active transport and metabolism. However, throughput, cost and availability of human tissues limit the routine use of this model. In order to integrate CYP3A4 activities into Caco-2 model, Takenaka et al. co-expressed recombinant human CYP3A4 and NADPH-CYP P450 reductase in Caco-2 cells [7]. Although a good correlation between extraction ratios observed in vitro and the gastro-intestinal (GI) extraction ratios in human could be observed for a number of reference compounds, the in vitro model tend to underestimate the GI extraction, indicating a rather low CYP3A4 activity in the model. Since CYP3A4 activities are readily detected in microsomes prepared from liver or intestine, Gertz et al. used another approach to improve the predictivity of firstpass extraction in human by combining the metabolic clearance of CYP3A4 compounds measured in human intestinal microsomes and the permeability data from Caco-2 or MDCK-MDR1 assay [8]. A clear disadvantage of this approach is that two separate in vitro measurements are needed. In recent years, new in vitro models for human intestine like microfluidic tissue-on-chip [9] or organoids [10] are emerging. These models have been shown as useful models for testing compound toxicity in GI tract or as disease models [9,10]. As an ADME model for human gut, however, a tissue model on a Transwell basis would be more favorable because the equipment for Caco-2 permeability assay could be easily adapted to the new model and would enable the measurement of transcellular permeability and transport in the new model. Two such models have been published recently with basic characterization regarding drug transporters and DMEs: The EpiIntestinal microtissues provided by MatTek [11] and the 3D bioprinted human intestinal tissues provided by Organovo [12]. Due to the earlier availability and easier accessibility we decided to evaluate the EpiIntestinal model as an ADME tool in more detail. The aim of the present study is the in-depth characterization of the EpiIntestinal microtissues as a model for the investigation of activities of drug transporters and DME and for the prediction of GI firstpass availability in human.

## 2. Materials and Methods

### 2.1. Material

All marketed drugs used in this manuscript and the metabolites and the deuterated standards thereof are purchased from commercial providers (Sigma, LKT laboratories, Roche, BD Gentest, Toronto Research, Cerilliant or Syncom). Dabigatran etexilate and its metabolites BIBR 951, BIBR 953, BIBR 1087 and internal research compounds of Boehringer Ingelheim are provided by the internal compound management.

### 2.2. Cell Culture

Caco-2 cells were obtained from Leibniz Institute DSMZ-German Collection of Microorganisms and Cell Cultures (Braunschweig, Germany) and cultured in DMEM containing 10% FCS, 1% NEAA, 2 mM Glutamin, 100 U/mL Penicillin and 100 µg/mL Streptomycin. Caco-2 cells were seeded either onto 24-well Transwell inserts (Corning, #3379) for bidirectional permeability assays or onto 96-well Transwell inserts (Corning #3391) for the screening of DME activities at a density of 160,000 cells/cm^2^ and cultured for 3 weeks, with media change on every second day. EpiIntestinal microtissues were obtained from MatTek (Bratislav, Slovakia) and cultured according to the manufacturer’s instruction (24-well format for bidirectional permeability assays and GI firstpass availability assays and 96-well format for screening of DME activities).

### 2.3. Bidirectional Permeability Assay

Bidirectional permeability assays were performed as described previously [13,14]. Briefly, compounds were diluted in transport buffer (128.13 mM NaCl, 5.36 mM KCl, 1 mM MgSO_4_, 1.8 mM CaCl_2_, 4.17 mM NaHCO_3_, 1.19 mM Na_2_HPO_4_, 0.41 mM NaH_2_PO_4_, 15 mM 2-[4-(2-hydroxyethyl)piperazin-1-yl]ethanesulfonic acid (HEPES), 20 mM glucose, pH 7.4) containing 0.25% bovine serum albumin to a final concentration of 10 µM and added to the apical or basal compartment. In indicated experiments, inhibitors were added to both compartments. Cells were incubated with the compounds for up to 2 h. Samples from the opposite compartment were taken at different timepoints. Compound concentrations in the samples were determined by HPLC-MS/MS (standard equipment: HPLC series 1000 or higher from Agilent, Santa Clara, CA, USA, and mass spectrometers API 4000 or higher from AB Sciex, Toronto, ON, Canada). Prior to bioanalysis samples were spiked with internal standard solution and diluted with acetonitrile (ACN) for protein precipitation. Measurement was operated in multiple reaction monitoring (MRM) mode. Quantification was performed using external calibration. Apparent permeability coefficients in the apical to basal direction (*P_app,AB_*) and in the basal to apical direction (*P_app,BA_*) and efflux were calculated as follows

Papp,AB=QAB(C0×s×t)

Papp,BA=QBA(C0×s×t)

Efflux=Papp,BAPapp,AB

where *Q* is the amount of compound recovered in the receiver compartment after the incubation time *t*, *C*_0_ the initial compound concentration given to the donor compartment, and s the surface area of the Transwell inserts. Efflux ratio is calculated as the quotient of *P_app,BA_* to *P_app,AB_*. As quality controls, one reference P-gp substrate (apafant) and one low permeable compound (BI internal reference, *P_app_* ≈ 3 × 10^−7^ cm/s, no efflux) is included in every assay plate. In addition, Transepithelial electrical resistance (TEER) values are measured for each plate before the permeability assay and total recovery in donor and receiver compartments was determined for each compound. All these parameters (efflux of apafant, *P_app_* values of the low permeable compound, TEER values, and total recovery) are used to ensure the quality of the assays.

### 2.4. Measurement of DME Activities in EpiIntestinal and Caco-2

For measurements of DME activities in EpiIntestinal microtissues and Caco-2 cells, both were cultured in 96-well Transwell inserts. Drugs (Table 1) were dissolved in the respective solvent at 200x concentration and diluted in a pre-warmed transport buffer. Diluted substrate solution was applied to the apical (100 µL) and basal (250 µL) compartment of the Transwell and incubated at 37 °C and 5% CO_2_ and 60 rpm continuous shaking. DME activities were determined by monitoring metabolite formation in basal compartment over time (0, 0.5, 1, 2, 3 and 4 h) with LC-MS/MS. For LC-MS/MS, an HTS-xt PAL autosampler (CTC Analytics), LC 1290 infinity G4220A (Agilent Technologies), column oven (Agilent Technologies) and 6500 TripleQuad (AB Sciex) were used. Chromatographic separation of samples was performed on YMC Triart C18 (1.9 µm, 30 × 2 mm; YMC Europe, Dinslaken, Germany) LC analytical column. Quantification of all metabolites listed in Table 1 was achieved by the use of calibration curves for the individual metabolites with appropriate concentration ranges.

A total of 10 µL of the incubation sample were diluted with 90 µL of water containing 10–20% ACN or methanol, 0.1% formic acid and the respective internal standard. To analyze the intracellular metabolite concentration, cells on Transwell inserts were washed twice with ice-cold PBS and stored at −80 °C for 20 min. Afterwards, 150 µL 50% ACN diluted with transport buffer was added to the cells and incubated at room temperature for 30 min. Cell lysate was transferred to a fresh 96-well plate and centrifuged at 4 °C, 4000 rpm for 10 min. Subsequently, 10 µL of the supernatant were diluted with 90 µl water containing 10–20% ACN or methanol, 0.1% formic acid and the respective internal standard. A total of 2 µL sample was injected into the LC-MS/MS system operated with an electrospray ionization source.

### 2.5. CES-Mediated Metabolism of Dabigatran Etexilate

CES-mediated metabolism of dabigatran etexilate was measured in cryopreserved human hepatocytes (BioIVT, West Sussex, UK) and cryopreserved human intestinal mucosa (in vitro ADMET laboratories, Columbia, MD, USA) in suspension and in Caco-2 cells and EpiIntestinal microtissues grown on Transwell inserts. Dabigatran etexilate was diluted in culture media for the respective cells or tissues. The final concentrations of dabigatran etexilate were selected in an earlier experiment to ensure reasonable turnover of the compound within the incubation time: 2 µM for hepatocytes and intestinal mucosa, 10 µM for Caco-2 and EpiIntestinal microtissues. In the experiments with Transwell inserts, dabigatran etexilate was given to the apical compartments, metabolites were measured in the basal (receiver) compartments. Concentrations of the metabolites were determined by HPLC-MS/MS (Section 2.3).

### 2.6. Metabolite Identification

Raloxifene or ezetimibe (10 µM) in culture media was added to the apical compartment of EpiIntestinal microtissues or incubated with cryopreserved human intestinal mucosa (HIM). Samples from basal compartment of EpiIntestinal microtissues or lysates of the incubation mixture with mucosa were prepared for metabolite identification as follows: samples were mixed with the same amount of 0.1% formic acid in ACN and subsequently evaporated and resuspended in water containing 25% methanol and 0.1% formic acid. Analysis was performed on a LC-MS system containing a Vanquish UPLC (ThermoFisher Scientific, San Jose, CA, USA) coupled to an Orbitrap FusionTribrid high resolution mass spectrometer (ThermoFisher Scientific). Structure elucidation was based on exact mass measurements in combination with the interpretation of fragment spectra.

### 2.7. Measurement of Intestinal First-Pass Availability in EpiIntestinal Microtissues and Caco-2

Compounds were diluted in culture media to a final concentration of 10 µM and added to the apical (donor) compartment (total volume: 100 µL for EpiIntestinal and 200 µL for Caco-2). After the incubation at 37 °C for 2, 4, 6, and 24 h, samples (50 µL) were taken from the basal (receiver) compartment (total volume: 5000 µL for EpiIntestinal and 800 µL for Caco-2). After the last timepoint, samples from the donor compartments and the cell lysates were also collected. Compound concentrations in the samples were determined by HPLC-MS/MS (Section 2.3).

GI first-pass availability of the tested compounds was expressed as fraction (%) of the total amount of a compound added to the donor compartment recovered in the receiver compartment.

### 2.8. Calculation of F_a_ × F_g_ in Human

The first-pass GI availability (*F_a_ × F_g_*) of selected drugs was calculated using the equation:

$$F_{a}\times F_{g}=F/F_{h}$$

where *F* is the total oral availability, *F_a_* the fraction absorbed, *F_g_* the intestinal availability and *F_h_* the hepatic availability. The hepatic availability *F_g_* can be estimated with the equation:

$$F_{g}=1-CL_{h}/Q_{H}$$

where *CL_h_* is the hepatic clearance of a drug and *Q_H_* the hepatic blood flow in human (20.7 mL/min/kg). The hepatic clearance *CL_h_* is calculated with the equation:

$$CL_{h}=CL_{b}\times \left(1-f_{e}\right)$$

where *CL_b_* is the blood clearance of a drug and *f_e_* the fraction of renal excretion. Blood *CL_b_* can be converted from plasma clearance *CL_p_* with the blood-to-plasma ratio *R_B_*:

$$CL_{b}=CL_{p}/R_{B}$$

A set of 12 marketed drugs are selected for the evaluation of EpiIntestinal microtissues. The clinical data for the calculation of *F_a_* × *F_g_* are summarized in Table 2.

## 3. Results

### 3.1. Barrier Function and Transporter Activities

EpiIntestinal microtissues as an intestinal permeability model have been tested in detail by Ayehunie et al. [11]. Our evaluation with in-house compounds and reference drugs (atenolol, fexofenadine, dabigatran, dabigatran etexilate, fenoterol, and otenzepad) showed similar data (not shown). However, the drug rosuvastatin which was used as a P-gp model drug by Ayehunie et al. [11] showed different results in our hands. Both in Caco-2 cells and in EpiIntestinal microtissues, the selective BCRP inhibitor Ko-143 (3 µM) strongly reduced the efflux of rosuvastatin, whereas the selective P-gp inhibitor zosuquidar (5 µM) only showed a minor effect on the efflux of rosuvastatin (Table 3). These data indicate BCRP as the major transporter in both in vitro models.

### 3.2. Drug-Metabolising Enzymes (DME) in EpiIntestinal Microtissues

**Figure 1:**
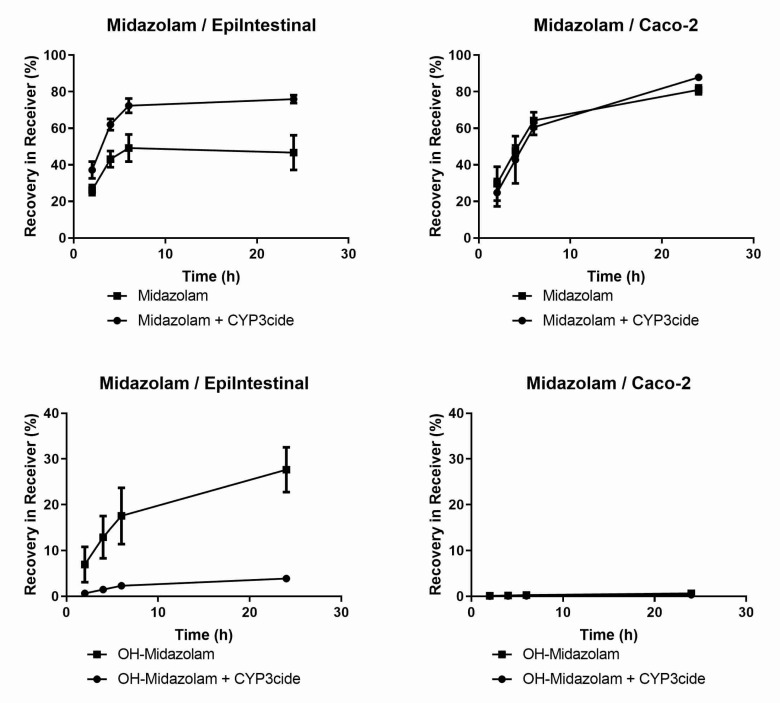
Apical-to-basal transport of midazolam in EpiIntestinal microtissues and Caco-2 cells. Midazolam (10 µM) was added to the apical compartment of EpiIntestinal microtissues (**left panels**) or Caco-2 cells (**right panels**) grown on Transwell inserts and incubated at 37 °C. At the time points as indicated, samples were taken from the basal (receiver) compartment. Midazolam (**upper panels**) and 1-Hydroxymidazolam (**lower panels**) were quantified in the samples via HPLC-MS/MS. The incubation was carried out in the absence or in the presence of the selective covalent CYP3A inhibitor CYP3acide (1 µM). Data are shown as mean values of triplicates. Error bars show standard deviations.

We studied the effect of DME on the GI first-pass availability with two model drugs: Midazolam for CYP3A4 [24] and astemizole for CYP2J2 [25,26]. As shown in Figure 1, midazolam showed a lower availability in EpiIntestinal microtissues than in Caco-2 (recovery in basal/receiver compartment after 24 h: 46.7% vs. 81.0%) when added to the apical compartment of both models. The addition of the covalent CYP3A4 inhibitor CYP3cide [27] increased the availability of midazolam in EpiIntestinal microtissues (46.7% vs. 75.9%), but had almost no effect on the availability in Caco-2 (81.0% vs. 87.9%). Moreover, substantial amount of 1-hydroxymidazolam, the CYP3A4-selective metabolite of midazolam, was detected in EpiIntestinal microtissues and was suppressed by the addition of the selective inhibitor, whereas only a negligible amount of the metabolite was detected in Caco-2, consistent with the low level of CYP3A4 expression in this cell line.

**Figure 2:**
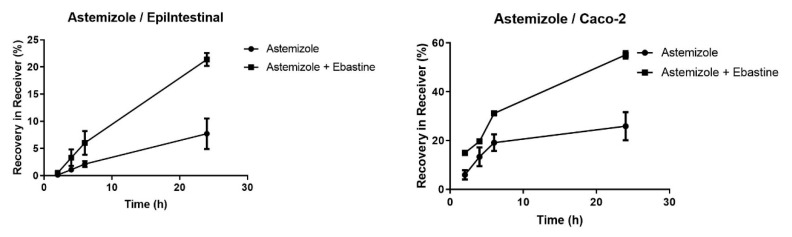
Apical-to-basal transport of astemizole in EpiIntestinal microtissues and Caco-2 cells. Astemizole (10 µM) was added to the apical compartment of EpiIntestinal microtissues (**left**) or Caco-2 cells (**right**) grown on Transwell inserts and incubated at 37 °C. At the indicated timepoints, samples were taken from the basal (receiver) compartment. Astemizole was quantified in the samples via HPLC-MS/MS. The incubation was carried out in the absence or in the presence of the competitive CYP2J2 inhibitor Ebastine (50 µM). Data are shown as mean values of triplicates. Error bars show standard deviations.

In contrast to CYP3A4, CYP2J2 activities were readily detected both in EpiIntestinal microtissues and in Caco-2 (Figure 2). In both models, co-incubation with the CYP2J2 inhibitor Ebastein (50 µM) [26] increased the availability of astemizole in the receiver compartment.

Encouraged by the results regarding CYP3A4 and CYP2J2 activities, we screened the activities of DME in EpiIntestinal microtissues more systematically: we quantified the formation of selective metabolite of the respective enzymes at different substrate concentrations (Figure S1). The substrate concentration approaching enzyme saturation (marked dots in Figure S1) was subsequently used to compare the activities in EpiIntestinal microtissues and Caco-2 cells. As shown in Table 4, the most prominent difference in DME activities between both models are the CYP3A4 activities, which could be demonstrated by two different substrates (testosterone and midazolam). In addition, CYP1A2 is the only enzyme with much lower activities in EpiIntestinal than in Caco-2 cells. Furthermore, we measured all metabolites also in the cell lysate. Some of the metabolites showed substantial intracellular accumulation. In order to monitor batch variability with regard to DME activities, experiments shown in Table 4 were repeated with microtissues from a different batch with comparable results (not shown).

### 3.3. Differential Expression of CES1 and CES2 in EpiIntestinal Microtissues and Caco-2 cells

**Figure 3:**
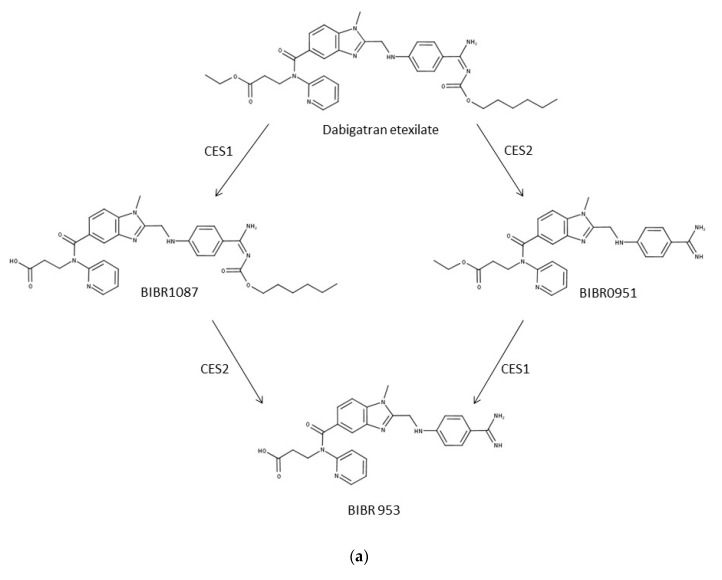

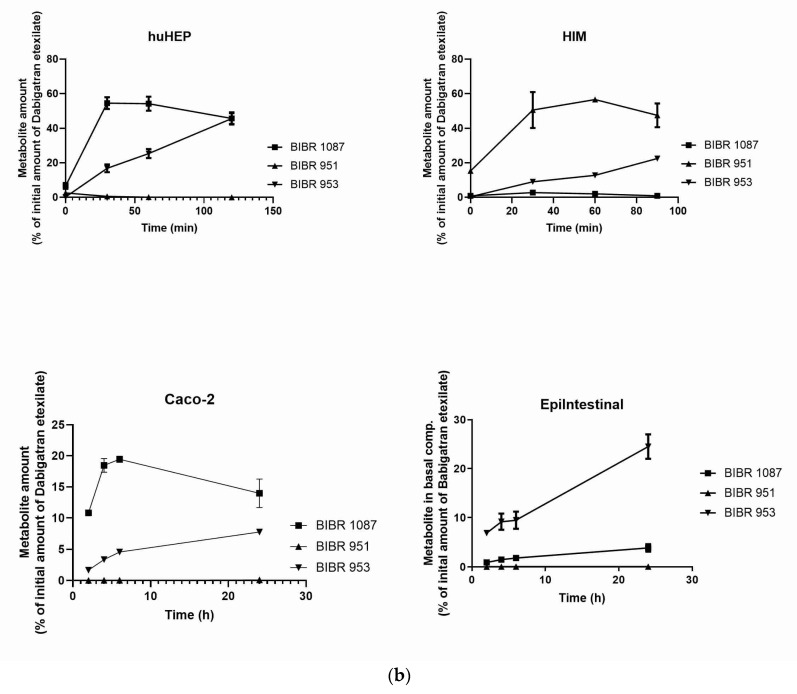
Metabolic pathways and metabolite pattern of dabigatran etexilate. (**a**) Metabolic pathways of dabigatran involving CES1 and CES2. (**b**) Detection of metabolites of dabigatran etexilate in human hepatocytes (huHEP), human intestinal mucosa (HIM), Caco-2 cells, and EpiIntestinal microtissues. Dabigatran was incubated with hepatocytes and intestinal mucosa in suspension, or given to the apical compartment of Caco-2 cells and EpiIntestinal microtissues and incubated at 37 °C. At the indicated timepoints, samples were taken from the suspension of huHEP and HIM or from the basal compartments of Caco-2 cells and EpiIntestinal microtissues. The metabolites were quantified using LC-MS/MS. Data shown as the mean and SD of triplicates.

Ishiguro et al. reported that Caco-2 cells, albeit originated from human colorectal carcinoma, resemble rather hepatocytes with regard to expression of the isoforms CES1 and CES2 [4]: Whereas CES2 is predominantly expressed in human intestine, CES1 is the major esterase in human hepatocytes and Caco-2 cells. In order to profile EpiIntestinal microtissues in this regard, we investigated the metabolism of dabigatran etexilate, the same substrate used by Ishiguro et al., in human hepatocytes (huHEP), cryopreserved human intestinal mucosa (HIM), Caco-2 cells and EpiIntestinal microtissues. The metabolic pathway for dabigatran etexilate is depicted in Figure 3a: the formation of the intermediate metabolite BIBR 1087 from the double prodrug dabigatran etexilate and the formation of the active drug from the intermediate metabolite BIBR 951 is catalyzed by CES1. The formation of BIBR 951 from dabigatran etexilate and the formation of the active drug BIBR 953 from BIBR 1087 is catalyzed by CES2. In tissues and cells with predominant expression of CES1 (liver e.g.,), BIBR 1087 should be the major metabolite; in tissues and cells with predominant expression of CES2, BIBR 951 should be the major metabolite (e.g., intestine). As shown in Figure 3b, in human hepatocytes and human intestinal mucosa, the expected metabolite pattern was observed—BIBR 1087 as the main metabolite in hepatocytes, and BIBR 951 as the main metabolite in human intestinal mucosa. Consistent with the data by Ishiguro et al., BIBR 1087 was found to be the main metabolite in Caco-2 cells. Interestingly, the metabolite pattern of dabigatran etexilate in EpiIntestinal microtissues resembles none of the other three models. The active drug BIBR 953 was found as the main metabolite in EpiIntestinal microtissues, suggesting similar CES1 and CES2 enzyme activities in this model.

### 3.4. UGT and SULT Activities in EpiIntestinal Microtissues

Extensive glucuronidation in the intestine is one of the major reasons that hamper the oral availability of drugs. Raloxifene and ezetimibe are two examples showing extensive intestinal glucuronidation in human [28,29,30]. When given to the apical compartment of EpiIntestinal microtissues, two putative glucuronides for both drugs were detected by mass scan (Table 5). We could confirm that these are glucuronides of both drugs by digestion with β-glucuronidase (data not shown). For ezetimibe, glucuronidation was the only metabolism found in EpiIntestinal microtissues. In case of raloxifene, we found an unexpectedly high amount of sulfation products of raloxifene (Table 5). To verify the physiological relevance of this finding, we performed metabolite identification for both drugs also with cryopreserved human intestinal mucosa. As shown in Table 5, the metabolite patterns of both drugs are similar in EpiIntestinal and in HIM: glucuronidation only for ezetimibe; glucuronidation and sulfation for raloxifene.

### 3.5. Prediction of F_a_ × F_g_ in Human using EpiIntestinal Microtissues

Since most of the relevant drug transporters and DMEs are present in EpiIntestinal microtissues, we were interested in finding out whether this model could serve as an in vitro model for the prediction of GI firstpass availability of drugs in human (*F_a_* × *F_g_*). For this purpose, we selected a panel of reference drugs with known human data and measured the recovery of these drugs in the basal compartment (equivalent to portal vene) after adding the drugs to the apical compartment (equivalent to GI lumen). The data are summarized in Table 6. We observed a good agreement between the recovery of the drugs in basal compartment of EpiIntestinal microtissues and the *F_a_* × *F_g_* in human.

## 4. Discussion

At present, the Caco-2 cell culture model is the most accepted in vitro model in the pharmaceutical industry for the estimation of drug absorption in human intestine. The lack of the major drug metabolizing enzyme CYP3A4, however, hampers the use of this model for the holistic understanding of drug absorption and metabolism during the first-pass GI transition. Recently, Ayehunie et al. described a new organotypic 3D human intestine model, the EpiIntestinal microtissues, with combined barrier/drug transproter functions and DME activities [11]. In this work, we could confirm the intact barrier function of the EpiIntestinal microtissues with transepithelial electrical resistance (TEER) measurement and with the permeability data of reference drugs and in-house compounds (data not shown). However, our data did not agree with the conclusion by Ayehunie et al. that rosuvastatin is a P-gp substrate. In our opinion, the discordance was mainly due to the different interpretation of the inhibition by Elacridar. Elacridar (GF120918) is a nonselective inhibitor for both BCRP [31] and P-gp [32]. At the concentration of 10 µM used by Ayehunie et al., a strong inhibition of both BCRP and P-gp can be expected. Thus, the inhibition of rosuvastatin efflux by Elacridar cannot be unequivocally attributed to P-gp inhibition. In contrast, the inhibitors we were using in this study are selective [31,33]. Our in-house evaluation showed that, at the concentrations we were using here (5 µM zosuquidar and 3 µM Ko-143), differential inhibition of P-gp and BCRP can be achieved. As shown in Table 3, only Ko-143 reduced in Caco-2 and EpiIntestinal rosuvastatin efflux strongly. Involvement of multiple transporters has been reported in the hepatobiliary transport of rosuvastatin, including OATP1B1, OATP1B3, OATP2B1, MRP2 (ABCC2), MDR1 P-gp (ABCB1), and BCRP (ABCG2) [34]. Except OATP1B1 and OATB1B3, all other transporters are expressed also in human intestine and Caco-2 cells [35,36,37]. In the clinic, however, only BCRP interaction has been related to increased bioavailability of rosuvastatin [38]. Our results here are in agreement with the clinical observation.

EpiIntestinal microtissues are an improved in vitro model for the intestinal barrier function. A clear advantage of this model over the Caco-2 cellular model is the physiologically relevant activities of CYP3A4 (Figure 1 and Table 4), which accounts for about 80% of total CYP content in human small intestine [39]. We could also detect activities of CYP2B6, CYP2C8, CYP2C9, 2C19, 2D6 and 2J2 in EpiIntestinal microtissues (Table 4), as reported for human intestine [39]. CYP1A2 activity, which is very low in human intestine, were detected in both in vitro models, with Caco-2 showing seven-fold higher activity (Table 4), suggesting that EpiIntestinal microtissues are closer to human intestine. Moreover, enzymes involved in phase 2 biotransformation (UGTs and SULTs) and carboxylesterases are present in EpiIntestinal microtissues at substantial levels (Table 4 and Table 5, Figure 3). It is important to note that we measured the respective metabolites of the tested reference drugs both in supernatant and in cell lysate. With the exception of amodiaquine and astemizole, the intracellular accumulation of metabolites was rather low. Since the intracellular accumulation of all measured metabolites is comparable in Caco-2 cells and in EpiIntestinal microtissues, we do not expect a bias in the relative comparison of enzyme activities between Caco-2 and EpiIntesitnal by measuring the metabolite in supernatant only (as shown in Table 4). In the case of carboxylesterase activities, we could demonstrate that EpiIntestinal microtissues are closer to human intestinal mucosa compared to Caco-2 cells, which resemble rather hepatocytes regarding relative CES1/CES2 activities. Because of the rather comprehensive expression of DMEs, EpiIntestinal microtissues can serve as a useful tool for the identification of intestine-specific metabolites, as we demonstrated with raloxifene and ezetimibe (Table 6). One surprising finding was the identification of sulfation of raloxifene not only in EpiIntestinal microtissue, but also in primary human intestinal mucosa. Mono- and diglucuronides were found in human plasma as major metabolites after oral administration; no other metabolites were identified (Prescription information for Evista, Eli Lilly). However, raloxifene was identified as a substrate for various SULTs and the sulfation of raloxifene occurs during incubation with cytosols from human liver and intestine [40,41] and in Caco-2 cells [42]. Moreover, raloxifene is reported to be a potent competitive inhibitor of sulfotransferase 2A1 (SULT2A1) with a K_i_ value very similar to its K_m_ value for SULT2A1 [41,43]. The sulfation of raloxifene we observed in EpiIntestinal microtissues was in line with the reported in vitro data in the literature and was obviously not due to a biased expression of SULTs in this model. A possible explanation for the missing raloxifene sulfate in human plasma could be a strong first-pass hepatic extraction and the subsequent excretion of the sulfate into bile.

Although cryopreserved primary human enterocytes and human intestinal mucosa are now available for the investigation of intestinal drug metabolism [44,45], the advantage of EpiIntestinal microtissues is the presence of both intact barrier function and comprehensive DME activities. The combined barrier function and DME activities in EpiIntestinal microtissues make it possible to evaluate intestinal first-pass availability (*F_a_* × *F_g_*) in humans in a single experiment. Indeed, the in vitro intestinal availability of 12 marketed drugs in EpiIntestinal microtissues (% recovery in receiver compartment) is in good agreement with *F_a_* × *F_g_* calculated from the clinical pharmacokinetic data of these drugs (Table 6). It is important to note that the in vitro availability in our model was obtained after an incubation time of 24 h, while the drug absorption in human intestine is usually completed after a few hours. The longer incubation time in the in vitro model can be mainly attributed to the higher ratio of drug amounts applied to the microtissues (1 nmol) to the surface area of the microtissues (0.6 cm^2^). The human small intestine mucosa, in contrast, has a surface area of 30 m^2^ [46]. The ratio of drug amounts to surface area is much lower. It would be interesting to compare human intestine tissues mounted in Ussing chambers with the EpiIntestinal microstissues in this regard. One would assume that the primary tissues would perform at least similarly to the EpiIntestinal model, and the low availability of suitable human tissues would limit the broader use of the primary materials in drug screening. There is however one caveat for using the EpiIntestinal model in this regard: the data are only meaningful if the quantities of DMEs and drug transporters in the model are comparable to the human intestine. Investigation into the expression of DMEs and drug transporters in EpiIntestinal microtissues is currently ongoing (transcriptomics) or planned (proteomics).

Although the EpiIntestinal microtissues provide a number of advantages compared to the currently available tools like Caco-2 cells, primary human enterocytes, or human intestinal mucosa, there are some limitations with regard to the use of the model in drug screening. One of the limitations is the unknown donor variability. According to the manufacturer, the microtissues we tested to date were derived from one single donor. For various reasons, we have not been able to get access to microtissues derived from other donors from the manufacturer to date. For drugs involving highly polymorphic metabolizing enzymes, data from a single donor are certainly not representative for the patient population. Therefore, it will be very important to investigate this model further in this regard in the future. Another limitation of this model is the static incubation conditions. Under physiological conditions, both the content in the intestine lumen and the blood at the basal side are under constant flow. The blood flow, for example, can reduce the diffusion of the drugs back into the enterocytes and thus limit the “recycling” of the drugs between blood and intestinal mucosa. Under static conditions, however, the recycling of the drugs might lead to an underestimation of the availability of the drugs, especially for those with extensive metabolic clearance in the intestinal mucosa. Due to the strong dilution effect in the apical-to-basal direction (100 vs. 5000 µL media volume in the apical and the basal compartment, respectively) we consider the effect of recycling, even under the static incubation, to be rather low. We tried to mimic the blood flow by replacing a large part of the media in the basal compartment with fresh media at the indicated sampling time points. The results were comparable to the static incubation (data not shown). Nevertheless, the integration of this model into a microfluidic system might still be interesting because this will make the combination with other organ models (e.g., liver-on-chip) possible.

In summary, our data here demonstrate that the EpiIntestinal microtissues are a useful tool for understanding drug absorption and metabolism in human intestine. The easy access of the model makes it very attractive for drug screening in the drug discovery process. It can also be used for the mechanistic understanding of intestinal drug–drug interaction or for the identification of intestine-specific metabolites.

## Figures and Tables

**Table 1:** Drugs applied for drug metabolizing enzymes (DME) activity screen.

Drug	DME/Metabolite	Internal Standard	Drug Concentration (µM)	Solvent
Phenacetin	CYP1A2/Acetaminophen	d4-Acetaminophen	10–100	20% ACN
Bupropion	CYP2B6/2-OH-Bupropion	d8-OH-bupropion	15–300	Aqua bidest.
Amodiaquine	CYP2C8/OH-Desethyl-Amodiaquine	d5-Desethylamodiaquine	20–200	Aqua bidest.
Diclofenac	CYP2C9/4-OH-Diclofenac	(13C6)4′-OH-Diclofenac	20–200	20% ACN
S-Mephenytoin	CYP2C19/4-OH-Mephenytoin	d3-OH-Mephenytoin	20–200	40% ACN
Testosterone	CYP3A4/6β-OH-Testosterone	d3-6β-OH-Testosterone	40–400	ACN/MeOH
Midazolam	CYP3A4/1-OH-Midazolam	d4-1-OH-Midazolam	5–100	Ready-to-use solution
Dextromethorphan	CYP2D6 Dextrorphan	d3-Dextrorphan	10–100	Aqua bidest.
7-OH-Coumarin	UGT/7-OH-Coumarin-Glucuronid	α-Naphtylglucuronid	15–150	40% ACN
7-OH-Coumarin	SULT/7-OH-Coumarin-Sulfat	α-Naphtylglucuronid	15–150	40% ACN
β-Estradiol	UGT1A1/β-Estradiol-3-Glucuronid	α-Naphtylglucuronid	20–200	DMSO
Astemizol	CYP2J2/O-Desmethyl-Astemizol	Dextrorphan tartrate	2–50	30% ACN + 10 mM HCl
BIBF1120	CES/BIBF1202	d8-BIBF1202	10–100	ACN/MeOH

**Table 2:** Clinical pharmacokinetic data of the selected drugs for the calculation of *F_a_* × *F_g_*. Data sources are indicated. Due to a lack of literature data or conflicting data in the literature, R_B_ values for several compounds are measured in-house (#).

Drug	F	R_B_	CL_p_ (mL/min/kg)	f_e_
Atenolol	0.5 [8]	0.95 #	2.5 [15]	1 [16]
Atorvastatin	0.14 [17]	0.85 #	8.93 [8]	0.01 [18]
Buspirone	0.05 [8]	0.81 [8]	28.3 [8]	0.45 [19]
Felodipine	0.15 [8]	0.7 [8]	11 [15]	0 [16]
Indinavir	0.6 [8]	0.84 [8]	18 [20]	0.085 [16]
Irinotecan	0.25 [8]	0.82 [21]	7 [15]	0.32 [16]
Midazolam	0.4 [8]	0.64 #	5.3 [15]	0 [16]
Nifedipine	0.9 [8]	0.67 [8]	7.3 [15]	0 [16]
Oxybutynin	0.06 [8]	0.686 [22]	5.1 [15]	0 *
Quinidine	0.9 [8]	0.87 [8]	4 [15]	0.15 [16]
Rosuvastatin	0.2 [8]	0.75 #	11 [23]	0.3 [23]
Saquinavir	0.04 [8]	0.74 [8]	13 [15]	0.01 *

* Prescription information at fda.gov.

**Table 3:** Inhibition of transporter-mediated efflux of rosuvastatin in Caco-2 and EpiIntestinal microtissues. Data are mean values of duplicates (Caco-2) or triplicates (EpiIntestinal microtissues).

Substrate	Inhibitor	Caco-2		EpiIntestinal	
		PappAB (10^−6^ cm/s)	Efflux	PappAB (10^−6^ cm/s)	Efflux
Rosuvastatin	None	0.3	21.0	0.3	100.0
	Ko-143 (3 µM)	0.5	5.5	2.6	3.1
	Zosuqidar (5 µM)	0.3	19.0	0.9	25

**Table 4:** Determination of activities of DMEs in EpiIntestinal microtissues and Caco-2 cells. Enzyme activities in EpiIntestinal microtissues and Caco-2 cells in 96-well Transwell plates were measured with the respective substrates shown in Figure S1 and at the marked concentration. Data shown as mean and SD from triplicates.

DME/Substrate	Caco-2		EpiIntestinal	
	Enzyme Activities * (pmol/h/cm^2^) Mean/SD	>Intracellular Metabolite (% of Total)	Enzyme Activities * (pmol/h/cm^2^) Mean/SD	Intracellular Metabolite (% of Total)
CYP1A2/Phenacetin	123.1/4.8	BLQ	17.4/3.0	BLQ
CYP2B6/Bupropion	BLQ	BLQ	2.6/0.9	BLQ
CYP2C8/Amodiaquine	11.2/1.9	36.5	107.9/49.1	37.5
CYP2C9/Diclofenac	20.8/1.6	12.8	28.4/1.6	14.7
CYP2C19/S-Mephenytoin	7.1/0.7	4.5	6.9/0.7	4.3
CYP3A4/Testosterone	26.5/3.6	BLQ	176.4/8.0	0.9
CYP3A4/Midazolam	BLQ	BLQ	1.9/0.5	14.9
CYP2D6/Dextromethorphan	10.9/2.7	BLQ	9.2/1.3	BLQ
UGT/7-OH-Coumarin	10,770.5/721.9	5.5	7583.4/855.2	10.0
SULT/7-OH-Coumarin	508.0/46.1	BLQ	1747.4/140.0	3.4
UGT1A1/β-Estradiol	65.3/6.9	3.2	243.3/6.7	2.8
CYP2J2/Astemizole	4.9/0.3	62.9	17.7/5.2	68.7
CES/BIBF 1120	370.9/31.4	24.7	400.0/12.9	13.3

* Determined as the rate of metabolite formation in supernatant; BLQ: Below limit of quantification.

**Table 5:** EpiIntestinal microtissues and HIM were incubated with 10 µM ezetimibe or raloxsifene. Metabolite scan was performed as described in “Materials and Methods”. Amount (Peak areas) of parent drugs and metabolites at the end of the incubation was expressed as percent of peak areas of parent drugs at the beginning of the incubation (T0).

Substrate	EpiIntestinal		Human Intestinal Mucosa (HIM)	
	Ezetimibe	Raloxifene	Ezetimibe	Raloxifene
Parent (% of parent drug at T0)	8.4	2.4	40.3	33.2
Glucuronides (% of parent drug at T0)	39.1	2.2	58.6	18.7
Sulfates (% of parent drug at T0)	n.d.	14.1	n.d.	2.0

n.d.: Not detectable.

**Table 6:** Comparison of GI firstpass availability measured in EpiIntestinal microtissues and *F_a_* × *F_g_* in human. GI firstpass availability in EpiIntestinal microtissues was determined as described in 2.7. *F_a_* × *F_g_* in human for the tested drugs was calculated from the clinical pharmacokinetic data, as described in Section 2.8.

Drug	Recovery in Basal Comp. @ 24h (%)	*F_a_* × *F_g_* in Human (%)	DMEs/Transporters
Atenolol	86	50	
Atorvastatin	43	61	CYP3A4/BCRP/MRP2
Buspirone	60	70	CYP3A4
Felodipine	47	62	CYP3A4
Indinavir	53	100	CYP3A4
Irinotecan	62	39	Esterases, CYP3A4
Midazolam	47	59	CYP3A4
Nifedipine	110	100	CYP3A4
Oxybutynin	16	9	Esterases, CYP3A4
Qunidine	85	100	CYP3A4, etc.
Rosuvastatin	30	62	CYP2C9/BCRP/MRP2
Saquinavir	18	25	CYP3A4/P-gp

## Supplementary Materials

The following are available online at https://www.mdpi.com/1999-4923/12/5/405/s1, Figure S1: Concentration dependence of activities of drug-metabolising enzymes in EpiIntestinal microtissues.
