# Supplementary material for: In-Depth Characterization of EpiIntestinal Microtissue as a Model for Intestinal Drug Absorption and Metabolism in Human
Source: Pharmaceutics. 2020 Apr 28;12(5):405. doi: 10.3390/pharmaceutics12050405 (PMC7284918; doi:10.3390/pharmaceutics12050405)
Supplement: Supplementary file 1 [file pharmaceutics-12-00405-s001.pdf]

pharmaceutics

Supplementary Materials: In-Depth Characterization of EpiIntestinal Microtissue as a Model for Intestinal Drug Absorption and Metabolism in Human

Yunhai Cui, Stephanie Claus, David Schnell, Frank Runge and Caroline MacLean

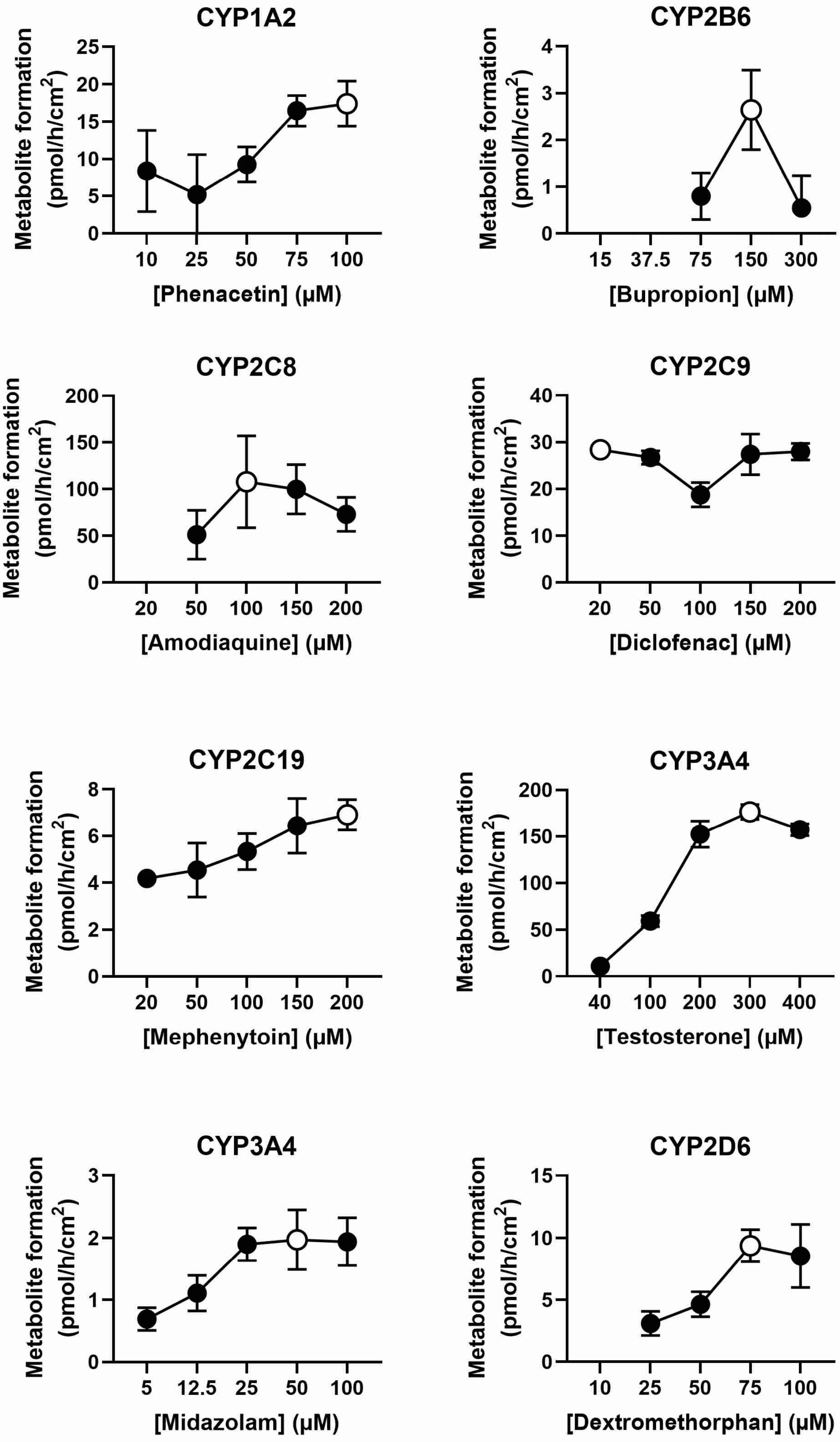
CYP1A2
Metabolite formation (pmol/h/cm2)
0 5 10 15 20 25
10 25 50 75 100
[Phenacetin] (μM)
CYP2B6
Metabolite formation (pmol/h/cm2)
0 1 2 3 4
15 37.5 75 150 300
[Bupropion] (μM)
CYP2C8
Metabolite formation (pmol/h/cm2)
0 50 100 150 200
20 50 100 150 200
[Amodiaquine] (μM)
CYP2C9
Metabolite formation (pmol/h/cm2)
0 10 20 30 40
20 50 100 150 200
[Diclofenac] (μM)
CYP2C19
Metabolite formation (pmol/h/cm2)
0 2 4 6 8
20 50 100 150 200
[Mephenytoin] (μM)
CYP3A4
Metabolite formation (pmol/h/cm2)
0 50 100 150 200
40 100 200 300 400
[Testosterone] (μM)
CYP3A4
Metabolite formation (pmol/h/cm2)
0 1 2 3
5 12.5 25 50 100
[Midazolam] (μM)
CYP2D6
Metabolite formation (pmol/h/cm2)
0 5 10 15
10 25 50 75 100
[Dextromethorphan] (μM)

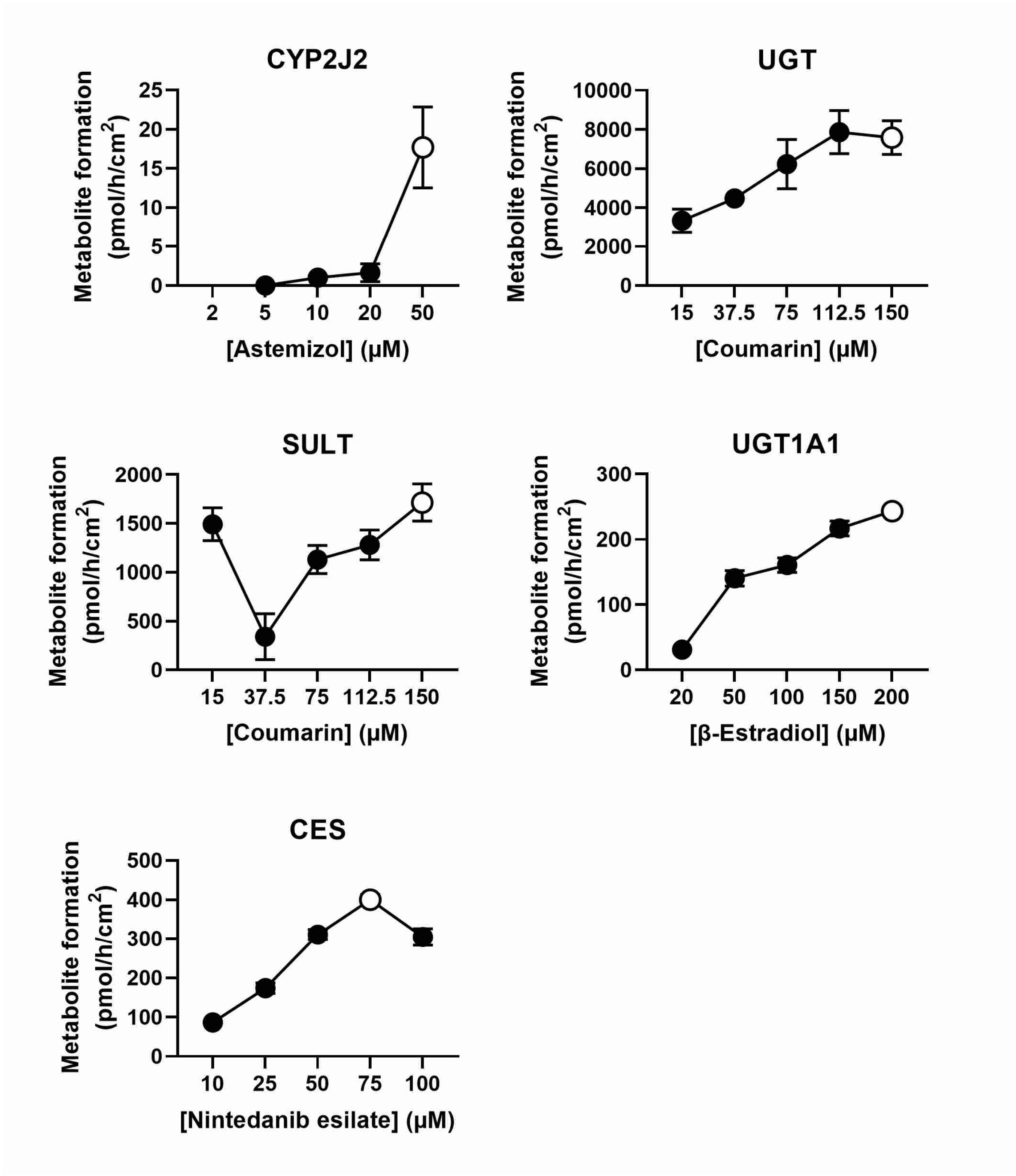

Figure S1. The concentration dependence of activities of drug-metabolising enzymes in EpiIntestinal microtissues. Metabolite formation by the respective drugs was measured as described in 2.4. Concentrations which were used for the comparison of the enzyme activities between EpiIntestinal microtissues and Caco-2 cells were marked with empty circles.
